# Versatile Roles of V-ATPases Accessory Subunit Ac45 in Osteoclast Formation and Function

**DOI:** 10.1371/journal.pone.0027155

**Published:** 2011-11-04

**Authors:** An Qin, Tak S. Cheng, Zhen Lin, Nathan J. Pavlos, Qing Jiang, Jiake Xu, Ke R. Dai, Ming H. Zheng

**Affiliations:** 1 Shanghai Key Laboratory of Orthopaedic Implant, Department of Orthopaedics, Shanghai Jiao Tong University School of Medicine, Ninth People's Hospital, Shanghai, The People's Republic of China; 2 Centre for Orthopaedic Research, School of Surgery, The University of Western Australia, Perth, Australia; 3 Division of Orthopaedic, Department of Surgery, Guangdong Academy of Medical Sciences, Guangdong General Hospital, Guangdong, The People's Republic of China; 4 Australian-China Joint Centre for Bone and Joint Research, Model Animal Research Centre of Nanjing University, Nanjing, The People's Republic of China; 5 The Center of Diagnosis and Treatment for Joint Disease, Drum Tower Hospital Affiliated to Medical School of Nanjing University, Nanjing, The People's Republic of China; 6 School of Pathology and Laboratory Medicine, The University of Western Australia, Perth, Australia; 7 Orthopaedic Cellular and Molecular Biology Laboratory, Institute of Health Sciences, School of Medicine, Chinese Academy of Sciences, Shanghai Jiao Tong University, Shanghai, The People's Republic of China; Ohio State University, United States of America

## Abstract

Vacuolar-type H^+^-ATPases (V-ATPases) are macromolecular proton pumps that acidify intracellular cargos and deliver protons across the plasma membrane of a variety of specialized cells, including bone-resorbing osteoclasts. Extracellular acidification is crucial for osteoclastic bone resorption, a process that initiates the dissolution of mineralized bone matrix. While the importance of V-ATPases in osteoclastic resorptive function is well-defined, whether V-ATPases facilitate additional aspects of osteoclast function and/or formation remains largely obscure. Here we report that the V-ATPase accessory subunit Ac45 participates in both osteoclast formation and function. Using a siRNA-based approach, we show that targeted suppression of Ac45 impairs intracellular acidification and endocytosis, both are prerequisite for osteoclastic bone resorptive function *in vitro*. Interestingly, we find that knockdown of Ac45 also attenuates osteoclastogenesis owing to a reduced fusion capacity of osteoclastic precursor cells. Finally, in an effort to gain more detailed insights into the functional role of Ac45 in osteoclasts, we attempted to generate osteoclast-specific Ac45 conditional knockout mice using a Cathepsin K-Cre-LoxP system. Surprisingly, however, insertion of the neomycin cassette in the Ac45-Flox^Neo^ mice resulted in marked disturbances in CNS development and ensuing embryonic lethality thus precluding functional assessment of Ac45 in osteoclasts and peripheral bone tissues. Based on these unexpected findings we propose that, in addition to its canonical function in V-ATPase-mediated acidification, Ac45 plays versatile roles during osteoclast formation and function.

## Introduction

The vacuolar-type H^+^-ATPase (V-ATPase) is a highly conserved plasma membrane protein complex that acidifies intracellular cargos and pumps protons across the plasma membranes in numerous cell types including osteoclasts, renal intercalated cells, epididymus and tumour cells [1]–[4]. The complex consists of more than 16 subunits that are divided into V1 and V0 domains [5]–[8]. The peripheral V1 domain is composed of eight subunits (A–H) driving ATP hydrolysis. Comparatively, the membrane bound V0 domain is comprised of six distinct subunits (*a*, *c*, *c′*, *c″*, *d* and *e*) with six copies of the *c*/*c′* subunits and single copies of *a*, *c″*, *d* and *e* subunits. The V0 domain utilizes the energy generated by the V1 domain to translocate protons across the membrane. While the structural diversity of the V-ATPase complex and tissue-specific isoforms have been shown to be associated with a multitude of cellular processes, the precise gamut of functions regulated by V-ATPases and their accessory subunits remain largely unclear.

Bone resorption by osteoclasts requires an ongoing secretion of acid to dissolve mineralized bone matrix. The macromolecular V-ATPase proton pump, located on the bone-apposed ruffled border membrane of osteoclasts, is an established prerequisite for proton secretion. Mutation, deletion or gene knockdown of different subunits of the V-ATPase complex in osteoclasts have been shown to severely impair osteoclastic bone resorption leading to severe osteopetrosis in both mice and man [3], [9]–[20]. Previously we and others have shown that up- regulation of the *d2* subunit of the V-ATPase complex is not only required for bone resorption but also facilitates osteoclast formation from committed precursors, pointing to auxiliary functions for selective V-ATPase subunits [13], [21].

Ac45 is an accessory subunit of the V-ATPase V_0_ complex originally isolated from bovine chromaffin granules and thought to participate in the rotational catalysis of the V0 domain [22], [23]. It exists as a globular protrusion of the V0 domain with its C-terminus anchored to the membrane and N-terminus projecting towards the luminal side evidenced by electron and cryo-electron microscopy [24]–[26]. In addition, the C-terminus of Ac45 carries a 26-amino acid (aa) residue domain that harbors an autonomous internalization signals that is important for the regulation of essential routing machinery and is necessary for efficient bone resorption by osteoclasts [10], [27].

To further explore the role of Ac45 in osteoclasts, we here employed an RNA interference strategy to specifically suppress Ac45 expression and investigate its impact on osteoclast formation and function. Interesting, we provide evidence that along with facilitating acidification and the up-take of endocytic markers, Ac45 also regulates osteoclast formation. In addition, we document the generation of osteoclast-specific Ac45 conditional knockout (cKO) mice**.** However, these mice unexpectedly exhibit marked disturbances in CNS development and ensuing embryonic lethality owing to the insertion of the neomycin cassette in Ac45-Flox^Neo^ mice thus precluding functional assessment of Ac45 in osteoclasts and peripheral bone tissues. Nonetheless, our collective *in vitro* findings highlight the remarkable yet versatile roles of Ac45 in osteoclast formation and bone resorption.

## Results

### siRNA-mediated knockdown of Ac45 impairs intracellular acidification, endocytosis and osteoclastic bone resorption *in vitro*

**Figure 1:**
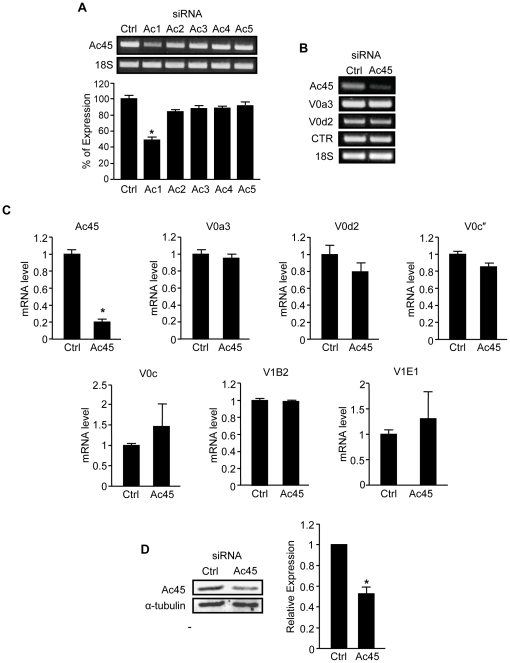
Efficiency and specificity of Ac45 siRNA knockdown in osteoclasts. (**A**) Five Ac45 siRNA oligonucleotides were tested for their ability to silence Ac45 mRNA expression. Semi-quantitative RT-PCR for Ac45 gene expression was carried out 48hrs post-transfection of siRNA oligos (100nM) into pre-osteoclasts. Expression levels were normalized to 18S and expressed as percentage of control ± SEM (n = 3). (**B**) Specificity and efficiency of Ac45 silencing. mRNA from pre-osteoclasts transfected with Ac45 siRNA for 48hrs was subjected to semi-quantitative RT-PCR analysis using specific primers for V-ATPase V0 domain subunits *a3* and *d2*, CTR and 18S. (**C**) Quantitative analysis of gene expression of Ac45 and V-ATPase V1 (B2 and E1) and V0 (*a3, d2, c″ and c*) subunits following Ac45 silencing using real-time qPCR. Data represented as relative mRNA level normalized to 36B4 control ± SEM (n = 3). (**D**) Ac45 protein level after Ac45 silencing in pre-osteoclasts for 48hrs. *P-values<0.05.

To investigate the function of Ac45 in osteoclasts we adopted a siRNA-based approach to specifically knock-down Ac45 expression in pre-fusion osteoclastic cells. Ac45 gene silencing was performed in bone marrow derived pre-fusion osteoclasts stimulated with 100 ng/ml RANKL for 3 days. Five different Ac45 siRNA oligonucleotides were initially screened to assess their ability to effectively silence Ac45 mRNA expression. As shown in Figure 1A, siRNA Ac1 proved the most effective in achieving a sustainable knockdown of Ac45 mRNA expression by more than 50% 48hrs post-transfection of pre-fusion osteoclasts which persisted upon multinucleated osteoclasts formation (day 5). The remaining siRNAs were deemed to have limited (<25%) to no effect on Ac45 mRNA expression levels (Fig. 1A) and thus excluded from use in subsequent experiments. Herein, the Ac1 siRNA is designated as Ac45 siRNA for subsequent investigations into the role of Ac45 in osteoclast differentiation and function *in vitro*. Pre-fusion osteoclasts were transfected with 100nM Ac45 or control siRNAs and cultured with RANKL and M-CSF for 48hrs to induce mature osteoclast formation, as evidenced by the presence of calcitonin receptor gene expression (Fig. 1B). In mature osteoclasts, following siRNA delivery, Ac45 mRNA and protein expression levels were reduced by 80% and 50% respectively as determined by real-time qPCR (Fig. 1B and C) and western blotting using a specific polyclonal antibody against Ac45 (Fig. 1D). It should be noted that newly synthesized Ac45 exists as precursor protein (ProAc45) of ∼60kDa that is subsequently cleaved to the mature functional form of ∼45-kDa (Fig. 1D) by proprotein convertase Furin (Fig. S1) [24], [28]. By comparison, gene expression levels of other known V-ATPase subunits including V0 subunits *a3*, *d2*, *c″* and *c*, and V1 subunits B2 and E1 remained constant following Ac45 siRNA-mediated silencing, attesting to the specificity of the siRNA (Ac1) to Ac45 (Fig. 1C).

An important function of V-ATPases in osteoclasts is to sustain the acidification of intramembranous organelles [29]. Therefore, as a first step towards investigating the function of Ac45 in osteoclasts we assessed the impact of Ac45 gene knockdown on osteoclastic intracellular acidification. To this end, we used complementary acridine orange fluorescence quenching (AO) and LysoSensor^TM^ Green DND-189 fluorescence detection assays to probe intracellular acidification in control and Ac45 knockdown osteoclasts [3], [13], [20], [30]–[32].

**Figure 2:**
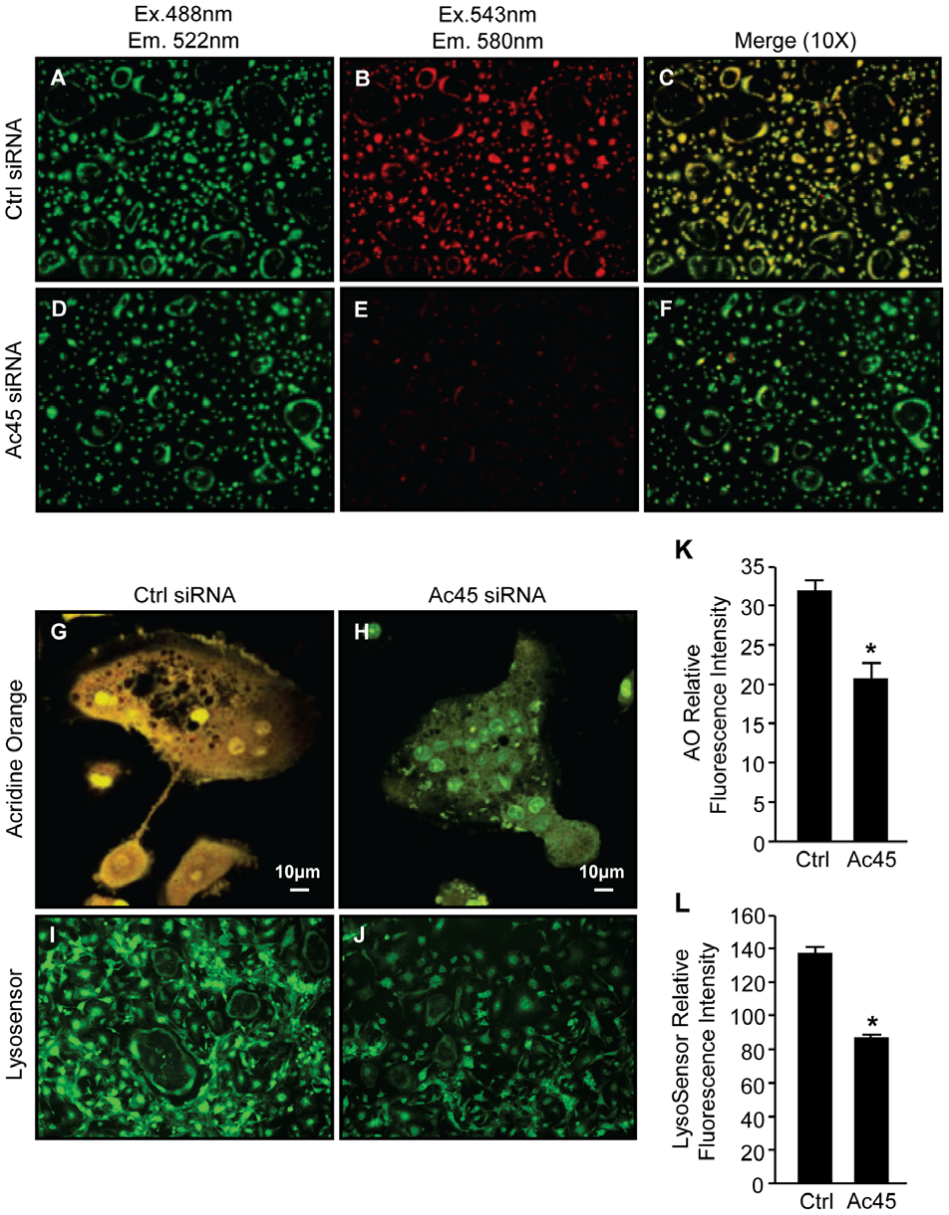
Ac45 is important for intracellular acidification in osteoclasts. Acridine orange (AO) staining of RANKL-stimulated BMM-derived osteoclasts after Ctrl siRNA (**A–C & G**) or Ac45 siRNA (**D–F & H**) treatment for 48hrs under 10X magnification. Osteoclasts transfected with siRNAs were incubated with 5 µg/ml AO for 30mins at 37°C followed by confocal microscopy. Cells were first excited with wavelength of 488nm and emission wavelength of 522nm for unprotonated AO detection (**A and D**), followed by excitation wavelength of 543nm and emission wavelength of 580nm for protonated AO detection (**B and E**). Fluorescence shift of acridine orange from green to red indicate normal intracellular acidification. (**C and F**) Image merge of red and green fluorescence spectra of Ctrl and Ac45 siRNA treated cells. (**G and H**) Higher magnification (40X) of merged fluorescence spectra of a representative osteoclast from Ctrl and Ac45 siRNA treated population. (**I and J**) Osteoclasts were treated with 1 µg/ml Lysosensor^TM^ Green DND-189 for 30min and excited at wavelength of 488nm. (**K and L**) The fluorescence intensity was quantified for both acridine orange and LysosensorTM Green DND-189 and expressed as relative fluorescence intensity (n = 3). *P-values<0.05.

As demonstrated in Figure 2D-F, Ac45-silenced precursors and mature osteoclasts exhibit little AO quenching evidenced by the predominantly green fluorescence upon overlay of the individual 488/543 fluorescence spectra. Little shift towards the red fluorescence was observed, reflecting reduced acidity in Ac45 knockdown cells. In contrast, cells transfected with control siRNA displayed typical intracellular acidification levels indicated by the fluorescence shift of AO fluorescence from green to red (Fig. 2A–C). Higher magnification (40X) and quantitative assessment further exemplifies the quenching of AO in control osteoclasts but not in Ac45-silenced osteoclasts as demonstrated by the green and red fluorescence spectral overlays (Fig. 2G, H and K). Additionally, LysoSensor^TM^ Green DND-189 further confirmed this intracellular acidification defect in Ac45 knockdown osteoclasts. In this instance, quantitative analysis of the LysoSensor^TM^ Green DND-189 fluorescence intensity (green) indicated that it was significantly reduced in Ac45-silenced osteoclasts as compared to the control siRNA transfected osteoclasts (Fig. 2I, J and L).

**Figure 3:**
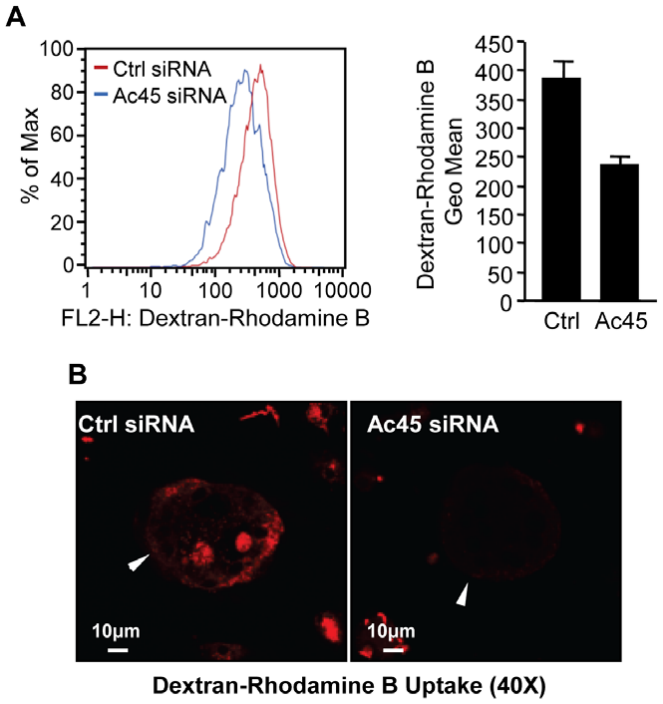
Ac45 mediates fluid-phase endocytosis in osteoclasts and their precursor cells. (**A**) Flow cytometric analysis of dextran-rhodamine B uptake by Ac45 siRNA treated BMM-derived osteoclasts and osteoclastic precursor cells. Cells transfected with siRNA oligos for 48hrs were treated with 50 µg/ml dextran-rhodamine B for 30mins followed by flow cytometric analysis. The percentage of cells with dextran-rhodamine B uptake was detected using 630/22nm bandpass with at least 10,000 events collected and analyzed to determine the proportion of cells containing dextran-rhodamine B and the geometric mean of the positive population. (**B**) Dextran-rhodamine B uptake by confocal microscopy. Osteoclasts treated with siRNA oligos were incubated with 50 µg/ml dextran-rhodamine B for 30mins prior to fixation for confocal analysis. Representative image of dextran-rhodamine B uptake by Ac45-silenced and control-treated osteoclasts from three independent experiments. Osteoclasts are indicated by the white arrowheads.

Because intracellular acidification by V-ATPases is also an important requirement for endocytosis uptake [5]–[8], [33], [34], we explored whether knockdown of Ac45 influenced the uptake of the endocytic marker dextran (fluid-phase endocytosis) in BMM-derived osteoclasts**.** Flow cytometric analysis revealed that knockdown of Ac45 correlated with a reduction in rhodamine B-conjugated dextran uptake when compared to that of the control group (Fig. 3A). This observation was confirmed by morphometric confocal assessment where significantly greater rhodamine B-dextran uptake was observed in osteoclasts from the control siRNA group as compared to those silenced for Ac45 (Fig. 3B).

**Figure 4:**
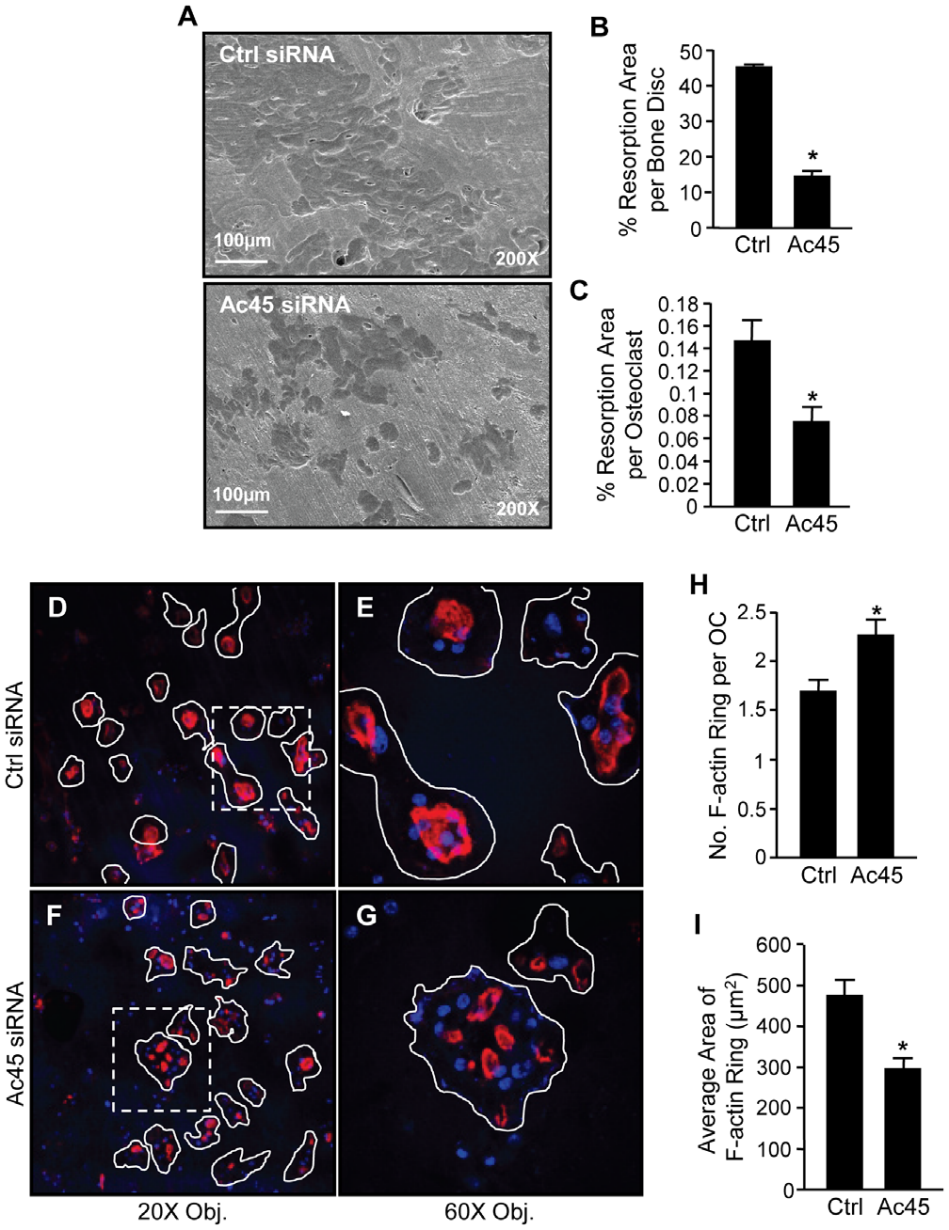
Ac45 is important for osteoclast bone resorption and F-actin ring formation. (**A**) Knockdown of Ac45 impairs bone resorption. Ac45-silenced pre-osteoclasts cultured on bone discs for 48hrs were examined for bone resorptive activity by scanning electron microscopy (SEM). (**B**) The area of bone resorptive pits in each bone disc was quantified as a percentage of the whole bone disc area. (**C**) The resorptive activity per osteoclast was quantified as the average bone resorption area per osteoclast (%; total area/total osteoclast number). *P-values<0.05. (**D and F**) Low magnification (20X) confocal analysis of F-actin ring formation in Ctrl and Ac45 siRNA treated osteoclasts cultured on bone discs. Ac45-silenced osteoclasts cultured on bone discs were fixed and stained with rhodamine-conjugated phalloidin for the visualization of F-actin. Nuclei were counterstained with Hoechst. (**E and G**) Higher magnification (60X) confocal analysis of dotted region in E and F respectively. Osteoclasts are outlined in white. The average number of F-actin ring per osteoclast (**H**) and the average area ( µm^2^) of F-actin rings (**I**) were quantified. Data representative of three independent experiments (mean ± SEM). *P-values<0.05.

Osteoclasts utilize V-ATPases clustered at the ruffled border to synthesis and secrete high concentrations of H^+^ protons into the resorbing compartment to remove of mineralized bone matrix (need reference here). Given the deficiencies observed in both intracellular acidification and endocytosis following Ac45 knockdown, two processes known to be essential for bone resorptive function, we analysed the effected of Ac45 suppression on the bone-resorptive capacity of osteoclasts. To this end, osteoclasts transfected with either Ac45 or control siRNAs were cultured on bone discs for 48hrs and analyzed for bone resorption by SEM. Osteoclastic bone resorptive activity was significantly impaired upon the suppression of Ac45 (Fig. 4A). Approximately two-third reduction in bone resorption area was observed in Ac45-knockdown osteoclasts as compared to controls (Fig. 4B). Furthermore, the average bone resorption area per osteoclast (total area/total osteoclast number) decreased by up to 50% in the Ac45-silenced group as compared to control (Fig. 4C). Surprisingly, however, despite the obvious resorptive defects, we found that Ac45 knockdown osteoclasts exhibited incomplete or intermediate F-actin rings when compared to control cells as visualized by Rhodamine-phalloidin staining (Figure 4 D–I). These intermediate F-actin rings were smaller in size and more numerous (Fig. 4H–I) and less well-organized per cell following Ac45 knockdown (compared Fig. 4E to Fig. 4G).

### Knockdown of Ac45 affects osteoclast formation and maturation

**Figure 5:**
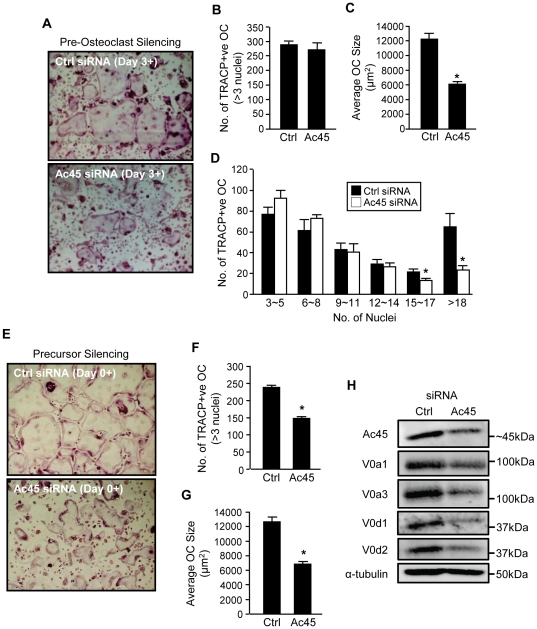
A role for Ac45 in osteoclast formation and fusion. (**A**) RANKL-stimulated pre-osteoclasts transfected with control or Ac45 siRNA for 48hrs were stained for TRAP activity to demonstrate multinucleated osteoclast formation. The number (**B**) and average size (µm^2^) (**C**) of TRAP+ve multinucleated osteoclasts (???3 nuclei) were quantified. (**D**) The number of nuclei per osteoclast from control and Ac45 siRNA treated groups. (**E–G**) BMM precursors were silenced for Ac45 expression prior to commission to the osteoclast lineage and sustained following RANKL stimulation for osteoclast formation. The number (**F**) and size (**G**) of resulting TRAP+ve osteoclasts were quantified. (**H**) Western blot analysis of protein expression of V-ATPase V0 domain subunits *a1*, *a3*, *d1*, and *d2* in osteoclasts following Ac45 gene silencing. Protein expression of α-tubulin was used as loading control. Data representative of three independent experiments (mean ± SEM). *P-values<0.05.

Along with their canonical role in acidification and bone resorption in osteoclasts, a subset of V-ATPase subunits have been recently implicated in non-canonical functions in osteoclast formation and maturation [13], [20]. Consistently, knockdown of Ac45 in pre-fusion osteoclasts stimulated with RANKL for 3 days led to a number of morphological aberrations in osteoclasts (Fig. 5A). Whereas the number of TRAP+ve multinucleated osteoclasts with 3 or more nuclei were similar between both Ac45 knockdown and siRNA control groups, (287±12.12 and 269±24.50 osteoclasts per well respectively) (Fig. 5B), the average area occupied by individual osteoclasts in the Ac45 siRNA group were significantly reduced compared to that of the control group (∼6000 µm^2^ compared to ∼12000 µm^2^ respectively) (Fig. 5C). Furthermore, examination of nuclei number per osteoclast revealed that siRNA-mediated knockdown of Ac45 led to a significant reduction in the number of osteoclasts that possessed >15 nuclei per cell (Fig. 5D, white bars) suggesting that suppression of Ac45 influences osteoclast fusion and/or maturation. Similar results were obtained when Ac45 gene expression was suppressed in BMM precursor cells prior to their commitment to the osteoclast lineage (Day 0) with Ac45 knockdown BMM precursor cells generating fewer and smaller TRAP+ve osteoclasts as compared with controls (6942±400 µm^2^ compared to 12138±700 µm^2^ respectively) (Fig. 5E–G). In an effort to identify a possible mechanism through which Ac45 silencing altered osteoclast formation, we further examined the protein expression of V0 domain subunits which have been implicated in membrane fusion [13], [20], [35]–[39] in Ac45 knockdown pre-osteoclastic cells. Interestingly, suppression of Ac45 expression substantially reduced the protein level of V0 subunits *a1*, *a3*, *d1* and *d2* (Fig. 5H), although the gene expression level was not changed (Fig. 1B and C), which may indicate a destabilization of the V0 domain complex. In addition, we also observed reduced protein expression levels of pro-fusogenic proteins such as *d2* and gene expression of ADAM8 (Fig. S2) which may also, at least in part, account for the reduced osteoclast formation and maturation phenotype observed given their previously assigned roles in membrane fusion [13], [40]. Collectively, the results suggest that Ac45 not only participates in canonical V-ATPase functions of acidification and bone resorption but also in non-canonical roles in osteoclast formation, fusion and maturation.

### Ac45- Flox^Neo^ mice exhibit embryonic lethality

**Figure 6:**
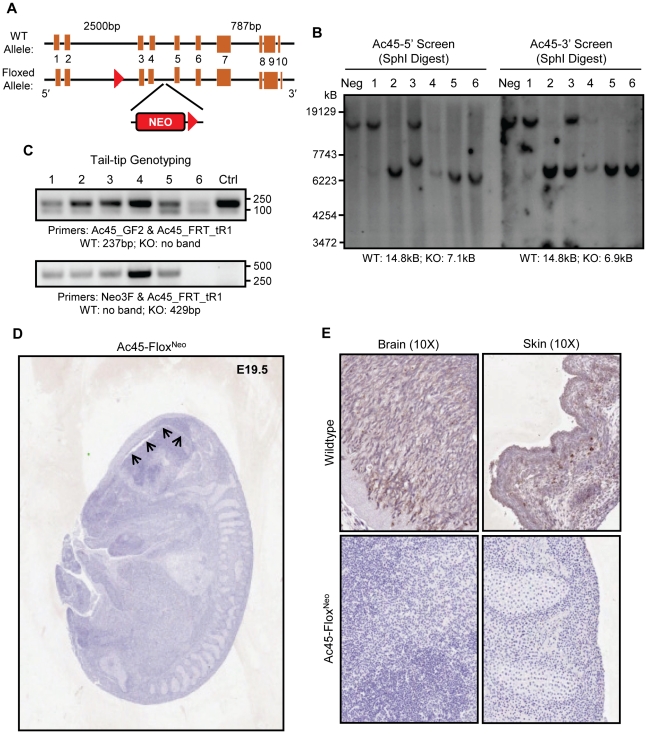
Ac45-Flox^Neo^ mice with neomycin is embryonic lethal. (**A**) Strategy of generating Ac45-Flox^Neo^ mice. The Ac45-Flox^Neo^ mice was generated by inserting two loxP sites in the intron upstream of exon 3 and another in the intron downstream of exon 4, with a neomycin-resistance (neo) expression cassette inserted as a selection marker. (**B**) Southern blot of six ES clones (1–6) and a negative control. 5′-SphI digest screening reveals WT and KO band size of 14.8 and 7.1kB respectively. 3′-SphI digest screening reveals WT and KO band size of 14.8 and 6.9kB respectively. (**C**) Tail-tip genotyping of the embryos demonstrates that clones 1 to 5 were Ac45-Flox^Neo^ embryo **(**Ac45_FRT_tR1 and Neo3F primers produce a product size of 429bp for floxed allele and no product for wildtype) while clone 6 is the WT embryo (Ac45_GF2 and Ac45_FRT_tR1 primers produce a product size of 237bp for wildtype and no product for floxed). (**D and E**) Immunohistochemistry demonstrated Ac45 expression is ubiquitous and high in brain and skin in the WT embryo whereas Ac45 expression was not detected in the Ac45-Flox^Neo^ embryo. In addition, the Ac45-Flox^Neo^ embryo had ventricular enlargement and neuronal loss, demonstrating impairment of the brain development (arrows).

Finally, in an effort to reconcile these *in vitro* observations and gain more precise insights into the role of Ac45 in osteoclasts, we attempted to generate osteoclast-specific conditional Ac45 knockout mice by cross-breeding Ac45-Flox^Neo^ mice with Cathepsin K-Cre knockin mice. For this purpose, a targeting vector floxing exons 3 and 4 of Ac45 (Fig. 6A) was electroporated into 129ES cells, followed by G418 and ganciclovir resistance selection. ES clones were screened for floxed alleles by PCR (data not shown) and southern blot analysis (Fig. 6B). Southern blot analysis identified clones 2, 5 and 6 as positive ES clones with the corresponding WT/KO band size of 14.8 and 7.1kB respectively using 5′-end and SphI digest screening, and 14.8 and 6.9kB respectively using 3-end and SphI digest screening (Fig.6B). The three positive ES clones were harvested for further blastocyst injection. Initial embryos implanted to pseudopregnant females failed to yield any viable mice due to embryonic lethality. Subsequent implantations resulted in six embryos that survived to E19.5. Tail-tip genotyping of these embryos identified embryos 1-to-5 as Ac45-Flox^Neo^ embryos with embryo 6 as WT (Fig. 6C).

Since global deletion of Ac45 has been previously reported to result in early embryonic lethality due to impaired blastocyst development [41], we probed the expression level of Ac45 in the Ac45-Flox^Neo^ embryos by immunohistochemistry. In WT embryos (E19.5) Ac45 was found to be ubiquitous and highly expressed in the brain and skin (Fig. 6E). On the other hand, Ac45 expression was not detected in the Ac45-Flox^Neo^ embryos (E19.5) (Fig. 6D and E). Histological analysis of whole mount Ac45-Flox^Neo^ E19.5 embryos further revealed severely compromised embryonic development, reminiscent of E12–13.5 WT embryos (as assessed in detail in [42], [43]) with impaired brain development, as demonstrated by ventricular enlargement and significant neuronal degeneration (Fig. 6D arrows and Fig. S3). While the precise reason for the embryonic lethality is presently unknown, these results provide further evidence for the importance of Ac45 in mouse embryonic development.

## Discussion

Accessory subunit Ac45 has been previously reported to co-purify with the V_0_ domain of the V-ATPase sub complex and thus is thought to constitute an integral component of the acidifying V-ATPase proton pump [22], [23]. Indeed, overexpression of Ac45 leads to increased granular acidification in Xenopus intermediate pituitary melanotrope cells attesting to its functional contribution to the V-ATPase acidification machinery [44]. In the present study, we demonstrate that knockdown of Ac45 impairs intracellular acidification in osteoclasts. In addition, suppression of Ac45 led to a significant reduction in fluid-phase endocytosis in osteoclastic cells suggesting that intracellular acidification is an important requirement for endocytic uptake and cargo processing. This position would be consistent with previous observations employing bafilomycin A1, a V-ATPase inhibitor, which blocked fluid-phase endocytosis in osteoclast precursor cells [45], [46]. Furthermore, we demonstrate that depletion of Ac45 inhibited the resorptive capacity of osteoclasts in vitro. Since osteoclastic bone resorption is dependent on V-ATPase-mediated extracellular acidification for the dissolution of the bone matrix as well as the endocytic uptake of degraded bone matrix from the resorption lacunae [34], [47]–[49], it is not surprising that knockdown of Ac45 resulted in impairments in osteoclastic bone resorption cycle. Together, our data lend support to the notion that, in osteoclasts, Ac45 plays a role in canonical V-ATPase-mediated processes including acidification and endocytosis and hence is an important requirement of osteoclastic bone resorption.

An interesting observation in the present study is that Ac45 knockdown osteoclasts exhibit more numerous and smaller intermediate/incomplete F-actin rings per osteoclast as compared to the control. The precise reason for this discrepancy is presently unclear. However, it is noteworthy to mention that similar cytoskeletal disruptions have also been recently reported in osteoclasts following the knockdown of the V-ATPase V1 domain subunit *C1* (ATP6V1C1) [50]. In this instance, the disruptions in F-actin ring formation appear to be independent of its role in V-ATPase activity given that the *C* subunit can bind and stabilize F-actin assembly autonomously [51], [52]. Thus it is conceivable that Ac45 plays a similar role in regulating F-actin ring assembly in osteoclasts. This position is further supported by our previous observation demonstrating that Ac45 colocalizes with the F-actin ring [10]. However, given that Ac45 knockdown decreased the protein levels of other V0 domain subunits which may destabilize the V0 domain complex, we cannot rule out the possibility that the effect on F-actin ring formation is independent of Ac45. Indeed, an intact V0 domain is a prerequisite for the assembly of the V1 domain with the V0 to form the V-ATPase complex. Thus the destabilization of the V0 may have impaired V1/V0 assembly and compromised V1-mediated F-actin ring formation. Future studies will be required to delineate these possibilities.

Another unexpected morphological effect observed following Ac45-depletion was the formation of smaller and fewer nucleated osteoclasts. These surprising observations imply that in addition to its role in the V-ATPase mediated acidification process; Ac45 may regulate non-canonical roles during osteoclastogenesis. Indeed, this phenotype is reminiscent of osteoclasts derived from the V-ATPase subunit *d2* knockout mice [13], [20]. In this case, genetic ablation of the *d2* subunit led to profound defects in pre-osteoclast fusion with no apparent intrinsic defect in osteoclast differentiation or V-ATPase activity [13]. Additionally, expression of other pro-fusogenic proteins such as DC-STAMP, ADAM8 and ADAM12 were also reduced in *d2* knockout cells [40], [53]. In line with this effect, Ac45 suppression markedly reduced the protein levels of *d2* and gene expression of ADAM8 and thus may also account for the reduced osteoclast formation and maturation phenotype observed. Moreover, there has been accumulating evidence to suggest that the V0 domain complex partakes in membrane fusion [35]–[39], however whether the entire V0 domain or specific subunits of the V0 is important for osteoclastic precursor fusion remains unclear. By comparison, loss or suppression of the V-ATPase *a3* and *C1* subunits demonstrate normal osteoclast differentiation and maturation potential, but exhibit acidification and bone resorption defects [3], [50]. Thus the current data suggests that specific V-ATPase subunits possess both canonical and non-canonical V-ATPase functions indicating that the role of V-ATPases in osteoclasts formation and function may be more complex than previously envisioned.

Finally, in an effort to elucidate the precise functional role that Ac45 in physiological bone homeostasis *in vivo*, we attempted to generate osteoclast-specific conditional knockout mice of Ac45. However, an unanticipated embryonic lethality of Ac45-Flox^Neo^ mice precluded the phenotypic assessment of Ac45 in osteoclasts of conditional knockout mice. This unexpected result highlights an important caveat that warrants consideration when designing Ac45 conditional knockout lines. While the precise reason for the embryonic lethality is currently under further investigation it appears likely that the inclusion of the neomycin cassette contributed to the lethality. In fact, recent studies indicate that inclusion of the neomycin cassette in an intron of various floxed genes can interfere with gene expression, compromising wildtype activity in the absence of Cre-mediated recombination [54]. This is presumably because the neomycin cassette contains cryptic splice sites that interfere with normal splicing and thus reduce wildtype mRNA levels [54], [55]. It is interesting to note that although our floxed mice survived to E19.5 of embryogenesis, the actual developmental stage resembles E12–13.5 embryos, as compared to the global knockout which impaired blastocyst development [41]. This provides some indication that Ac45 was likely expressed at an earlier point of embryonic development but that the Ac45 levels were subsequently diminished possibly owing to neomycin interference and thus was unable maintain Ac45 expression levels required for the survival of the embryo at later stages of development. This may also account for the discrepancy in our observations when compared to the global knockout where targeted disruption of Ac45 occurred at the ES cell stage. Thus to circumvent this problem and generate an osteoclast-specific Ac45 knockout, an alternative strategy would be to use three loxP sites to flank the neomycin cassette and the region of interest of Ac45 gene. A partial Cre-mediated recombination can be carried out to remove the neomycin cassette, leaving the region of interest of the Ac45 gene floxed [56]. This approach is currently underway in our laboratory and should hopefully yield further insights into this perplexing V-ATPase accessory subunit.

In conclusion, the results of this study indicate that accessory subunit Ac45 is an integral component of the osteoclast V-ATPase machinery, regulating multiple facets of V-ATPase function including canonical roles of acidification, endocytosis and bone resorption as well as and non-canonical roles in osteoclast formation and maturation, suggesting that Ac45 is an important yet remarkably versatile regulator of V-ATPase function.

## Materials and Methods

### Reagents and Antibodies

GST-RANKL_160-318_ (rRANKL) recombinant proteins were expressed and purified in our laboratory as previously described [57]. Mouse Ac45 silencing RNA (siRNA) oligonucleotides were purchased from Santa Cruz Biotechnology, Dharmacon Inc. (Thermo Scientific), and Shanghai GenePharma Co. Ltd. HiPerfect and Lipofectamine2000 transfection reagent were purchased from QIAGEN and Invitrogen respectively. Alexa Fluor 647 Phalloidin, Dextran-Rhodamine B (10000MW), and Hoechst 33258 were purchased from Invitrogen. Acridine orange was purchased from Sigma, LysoSensor^TM^ Green DND-189 probe was purchased from Invitrogen, and Geneticin G418 sulfate was purchased from Gibco. Rabbit polyclonal anti-Ac45 antibody raised against the last 12-aa residues of mouse Ac45 was kindly provided by Prof. E. Jansen from the Department of Molecular Animal Physiology, University of Nijmegen, Netherlands [24]. The anti-Ac45 antibody was pre-cleared as previously described [10]. Affinity purified rabbit polyclonal anti-*d2* antibody was raised against GST-*d2* peptide antigen produced in our lab [21].

### Cells and Cell Culture

Bone marrow monocytes (BMM) isolated from long bones of 6-weeks old C57BL/6 mice were cultured in complete α-modified Eagle's medium (αMEM) (10% v/v fetal bovine serum (FBS), 2mM L-glutamine, 100u/ml penicillin, 100 µg/ml streptomycin) supplemented with 10ng/ml M-CSF. Adherent M-CSF-dependent osteoclast precursor cells were stimulated with 100ng/ml rRANKL in the presence of 10ng/ml M-CSF for 5 days for the formation of multinucleated osteoclasts. Cells positive for TRAP activity and contained 3 or more nuclei were scored as mature osteoclasts.

### Semi-quantitative Reverse Transcription (RT)-PCR and Real-time qPCR

Total cellular RNA was isolated from cultured cells using RNeasy Mini Kit (QIAGEN) in accordance with the manufacturer's protocol. For RT-PCR, single-stranded cDNA was reverse transcribed from 2 µg total RNA using reverse transcriptase with oligo-dT primer. All PCR was carried out using 2 µl of each cDNA using the following cycling parameters 94°C, 40secs; 60°C, 40secs; and 72°C, 40secs for 30 cycles with primers shown in Table S1. PCR samples were analyzed by DNA agarose gel electrophoresis. For real-time qPCR analysis, corresponding PCR primers were used as shown in Table S1 and performed on a 384-well plate ABI Prism 7000 Sequence Detection system (Applied BioSystems, USA) using SYBR Green PCR Master Mix (QIAGEN). Cycling conditions was as follows 95°C, 15secs; 60°C, 20secs; and 72°C, 1min for 40 cycles. The comparative 2^−ΔΔCT^ method was used to calculate the relative expression of each target gene as previously described [58] and the expression level of all genes were normalized to the expression level of the house keeping gene 36B4.

### Western Blot

Total cellular proteins were extracted from cultured cells using RIPA RIPA lysis buffer (50mM Tris pH7.5, 150mM NaCl, 1% v/v Nonidet P-40, 0.1% SDS, 1% sodium deoxycholate) supplemented with Protease Inhibitor Cocktail (Roche). Lysates were cleared by centrifugation at 16,000g for 10mins at 4°C and supernatant containing proteins were collected. For immunoblotting, extracted proteins diluted in SDS-sampling buffer were resolved by SDS-PAGE (10–15%) gels and then electroblotted onto nitrocellulose membranes (Hybond ECL, Amersham Life Science). Following transfer, membranes were blocked with 5% w/v skim milk in TBS-Tween (TBS; 0.05M Tris, 0.15M NaCl, pH7.5 and 0.2% v/v Tween-20) for 1hr and then probed with primary antibodies diluted in 1% w/v skim milk powder in TBS-Tween for 2hrs. Membranes were washed in TBS-Tween and then incubated with HRP-conjugated secondary antibodies and antibody reactivity was detected by the Enhanced Chemiluminescence (ECL) detection system (Amersham Biosciences) using the FujiFilm LAS-3000 Gel Documentation System (FujiFilm) and its associated software.

### Silencing RNA Oligonucleotide Transfection

Ac45 and control (scrambled) siRNA oligos were transfected into BMM-derived pre-osteoclasts (after 3 days 100ng/ml rRANKL stimulation) to a final concentration of 100nM using HiPerfect transfection reagent (QIAGEN) according to our previously established protocol [59] . Gene knockdown efficiency was validated 48hrs post-transfection by RT-PCR analysis as described previously. Silenced osteoclasts were processed for bone resorption, immunofluorescence, intracellular acidification, and dextran uptake assays.

### Intracellular Acidification

Intracellular acidification was determined by acridine orange (AO) quenching and lysosensor fluorescence staining assays. Silenced osteoclasts were serum-starved for 2hrs followed prior to incubation with 5 µg/ml AO (Sigma) or 1 µg/ml lysosensor^TM^ green DND-189 for 30mins at 37°C. Cells were washed and processed for confocal microscopy analysis on a NIKON A1Si spectral detector confocal system under 10X and 40X lenses. For acridine orange quenching, cells were first excited with wavelength of 488nm and emission wavelength of 522nm (unprotonated AO detection), followed with excitation wavelength of 543nm and emission wavelength of 580nm (protonated AO detection). Fluorescence shift of acridine orange from green to red indicates normal intracellular acidification. For lysosensor^TM^ green DND-189 fluorescence detection, cells were excited at wavelength of 488nm (lysosensor^TM^ green DND-189 will fluoresce when inside acidic compartments and but no fluorescence can be detected if it is inside neutral compartments). The signal intensity was measured using the software accompanying the NIKON A1Si spectral detector confocal system.

### Fluid-Phase Dextran Uptake

BMM-derived pre-osteoclasts transfected with siRNA oligos for 48hrs were serum-starved for 2hrs followed by incubation with 50 µg/ml of Dextran-rhodamine B. Cells were washed and processed for flow cytometric and/or confocal microscopic analysis. Dextran-rhodamine B uptake by cells were analyzed on a Becton Dickinson FACS Vantage (San Jose, California) instrument with a 488nm laser in the primary position and a 568nm laser in the secondary position. Dextran-rhodamine B was detected using a 630/22nm bandpass filter in front of FL5. Sample files containing at least 10,000 events were collected using CellQuest software (Version 3.1f, Becton Dickinson, San Jose, California) and analyzed to determine both the proportion of cells containing Dextran and the geometric mean of the positive population.

### F-actin ring Immunofluorescence

For F-actin ring immunofluorescent staining, silenced osteoclasts cultured on bovine bone discs were fixed with 4% paraformaldehyde for 15min at room temperature (RT) and permeabilized for 5mins with 0.1% v/v Triton X-100. Cells were incubated with rhodamine-conjugated phalloidin (1∶100; Invitrogen) diluted in 0.2% w/v BSA-PBS for 1hr at RT and washed extensively with 0.2% w/v BSA-PBS and PBS. Cells were then incubated with Hoechst 3342 dye (1∶5000; Invitrogen) for visualization of nuclei, washed with PBS and mounted with ProLong Gold anti-fade mounting medium (Invitrogen). Detection of fluorescence was carried out on the NIKON A1Si spectral detector confocal system equipped with 20X (dry) and 60X (oil) lenses. Fluorescence images were collected using the systems NIS-C Elements software and analysed using ImageJ.

### Bone Resorption assay

Pre-osteoclasts seeded on bovine bone discs were silenced for Ac45 gene expression as described above. Forty-eight hours post-transfection, bone discs were fixed in 4% paraformaldehyde and stained for TRAP activity. The number of TRAP+ve multinucleated osteoclasts were scored prior to assessment of resorptive activity. Resorption pits were visualized by scanning electron microscopy. ImageJ (NIH) software was used to quantify bone resorption parameters.

### Generation of Ac45 conditional knockout mice

The Ac45 conditional allele (Ac45-Flox^Neo^) was generated by inserting loxP sites in the intron upstream of exon 3 and in the intron downstream of exon 4, with a neomycin-resistance (neo) expression cassette inserted as a selective marker for the ES cells (Fig.1A). Cre-mediated deletion of the flanked sequence, would delete exon 3 and 4 leading to frameshift mutation a null allele. After construction of the targeting vector from a B6 BAC clone, the plasmid was linearized by PacI digestion and eletroporated into 129ES cells. The transformants were selected for their G418 and ganciclovir (Ganc) resistance. 3′-end screening of ES cell DNA by PCR using primers Ac45-C3R3: ACCTGTCCCCAGCACAATCACATCG; and Neo3F: TCTGAGGCGGAAAGAACCAG; to produce a band 4.8kb for heterozygous flox/wt and no band for wt/wt; 5′-end screening with primers: Ac45_loxp_tF1: TTGTTCTTTGCTGTCCTCTTCCT and AC45_loxp_tR1: CAGGCTAACAAAAGAGTGAACCATC; produced band of 497bp for heterozygous flox/wt and 231bp for wt/wt. ES clones were subsequently screened by southern blot analysis using 5′-SphI (WT/KO band size of 14.8 and 7.1kB respectively) and 3′-SphI (WT/KO band size of 14.8 and 6.9kB respectively) digest screening. Flox^Neo+ve^ ES clones were subsequently injected into blastocyst and transferred to pseudopregnant C57BL/6 ICR female mice. Tail-tip genotyping was carried out with primers Ac45_FRT_tR1: GGGCATTAGTGGTAACAAAGTTTTCTC and Neo3F: TCTGAGGCGGAAAAGAACCAG to produce a product size of 429bp for floxed allele and no product for wildtype; and Ac45_GF2: AGCCTCTAACCTTGCACCTCAG and Ac45_FRT_tR1 above to produce a product size of 237bp for wildtype and no product for floxed. This study was carried out in strict accordance with the recommendations contained within the Australian Code of Practice for the Care and Use of Animals for Scientific Purposes. The protocol was approved by the Animal Ethics Committee (AEC) of the University of Western Australia (Permit ID: RA/3/100/865).

### Histology and Immunohistochemistry assessment of Ac45-Flox^Neo^ Embryo

For morphological analysis, embryo specimens were fixed and embedded in paraffin wax and then sectioned at a thickness of 5 µm according to standard procedures. For immunohistochemistry, antigen-retrieval using microwave heating was performed. Primary antibodies against mouse Ac45 were used at a dilution of 1∶1000. Standard immunohistochemistry protocols were performed while positive and negative controls were routinely included.

### Statistics & Data Presentation

Results were statistically analyzed using a two-tailed Students T-test using Microsoft Excel (Microsoft Corp.) and data shown represent one of at least three independent experiments

## Supporting Information

Figure S1 **ProAc45 protein level following Ac45 silencing.** Following 48hrs transfection with 100nM siRNA, total cellular proteins were extracted from transfected mBMM as described in Materials and Methods. Rabbit polyclonal anti-Ac45 antibody was used to detect proAc45 and mature Ac45 [24]. Ac45 is produced as a ∼62kDa precursor protein (proAc45) that is subsequently processed into the mature ∼45kDa form. The Ac45 siRNA appears to affect both the proAc45 and mature Ac45 protein levels.
(TIF)

Figure S2 **Quantitative analysis of Adam8 and Adam12 gene expression following Ac45 silencing using real-time qPCR.** mRNA from pre-osteoclasts transfected with Ac45 siRNA for 48hrs was subjected to qPCR analysis using specific primers for ADAM8 and ADAM12. Data represented as relative mRNA level normalized to 36B4 control ± SEM (n = 3).
(TIF)

Figure S3 **Comparison of WT wildtype E19.5 embryos and Ac45-Flex^Neo^ embryo.** H&E staining demonstrated that well-developed brain structure in the wildtype E19.5 embryos whilst the brain in Ac45-Flex^Neo^ embryo development is severely impaired.
(TIF)

Table S1 **Primers for Semi-quantitative and Real-time PCR.**
(DOCX)

## References

[1] Smith AN, Skaug J, Choate KA, Nayir A, Bakkaloglu A (2000). Mutations in ATP6N1B, encoding a new kidney vacuolar proton pump 116-kD subunit, cause recessive distal renal tubular acidosis with preserved hearing.. Nat Genet.

[2] Martinez-Zaguilan R, Lynch RM, Martinez GM, Gillies RJ (1993). Vacuolar-type H(+)-ATPases are functionally expressed in plasma membranes of human tumor cells.. Am J Physiol.

[3] Li YP, Chen W, Liang Y, Li E, Stashenko P (1999). Atp6i-deficient mice exhibit severe osteopetrosis due to loss of osteoclast-mediated extracellular acidification.. Nat Genet.

[4] Frattini A, Orchard PJ, Sobacchi C, Giliani S, Abinun M (2000). Defects in TCIRG1 subunit of the vacuolar proton pump are responsible for a subset of human autosomal recessive osteopetrosis..

[5] Forgac M (2007). Vacuolar ATPases: rotary proton pumps in physiology and pathophysiology.. Nat Rev Mol Cell Biol.

[6] Toei M, Saum R, Forgac M (2010). Regulation and isoform function of the V-ATPases.. Biochemistry.

[7] Forgac M (2000). Structure, mechanism and regulation of the clathrin-coated vesicle and yeast vacuolar H(+)-ATPases.. J Exp Biol.

[8] Stevens TH, Forgac M (1997). Structure, function and regulation of the vacuolar (H+)-ATPase.. Annu Rev Cell Dev Biol.

[9] Hu Y, Nyman J, Muhonen P, Vaananen HK, Laitala-Leinonen T (2005). Inhibition of the osteoclast V-ATPase by small interfering RNAs.. FEBS Lett.

[10] Feng H, Cheng T, Pavlos NJ, Yip KH, Carrello A (2008). Cytoplasmic terminus of vacuolar type proton pump accessory subunit Ac45 is required for proper interaction with V(0) domain subunits and efficient osteoclastic bone resorption.. J Biol Chem.

[11] Laitala T, Vaananen HK (1994). Inhibition of bone resorption in vitro by antisense RNA and DNA molecules targeted against carbonic anhydrase II or two subunits of vacuolar H(+)-ATPase.. J Clin Invest.

[12] Laitala-Leinonen T, Lowik C, Papapoulos S, Vaananen HK (1999). Inhibition of intravacuolar acidification by antisense RNA decreases osteoclast differentiation and bone resorption in vitro.. J Cell Sci.

[13] Lee SH, Rho J, Jeong D, Sul JY, Kim T (2006). v-ATPase V0 subunit d2-deficient mice exhibit impaired osteoclast fusion and increased bone formation.. Nat Med.

[14] Scimeca JC, Quincey D, Parrinello H, Romatet D, Grosgeorge J (2003). Novel mutations in the TCIRG1 gene encoding the a3 subunit of the vacuolar proton pump in patients affected by infantile malignant osteopetrosis.. Hum Mutat.

[15] Sobacchi C, Frattini A, Orchard P, Porras O, Tezcan I (2001). The mutational spectrum of human malignant autosomal recessive osteopetrosis.. Hum Mol Genet.

[16] Sundquist K, Lakkakorpi P, Wallmark B, Vaananen K (1990). Inhibition of osteoclast proton transport by bafilomycin A1 abolishes bone resorption.. Biochem Biophys Res Commun.

[17] Sundquist KT, Marks SC (1994). Bafilomycin A1 inhibits bone resorption and tooth eruption in vivo.. J Bone Miner Res.

[18] Taranta A, Migliaccio S, Recchia I, Caniglia M, Luciani M (2003). Genotype-phenotype relationship in human ATP6i-dependent autosomal recessive osteopetrosis.. Am J Pathol.

[19] Visentin L, Dodds RA, Valente M, Misiano P, Bradbeer JN (2000). A selective inhibitor of the osteoclastic V-H(+)-ATPase prevents bone loss in both thyroparathyroidectomized and ovariectomized rats.. J Clin Invest.

[20] Wu H, Xu G, Li YP (2009). Atp6v0d2 is an essential component of the osteoclast-specific proton pump that mediates extracellular acidification in bone resorption.. J Bone Miner Res.

[21] Feng H, Cheng T, Steer JH, Joyce DA, Pavlos NJ (2009). Myocyte enhancer factor 2 and microphthalmia-associated transcription factor cooperate with NFATc1 to transactivate the V-ATPase d2 promoter during RANKL-induced osteoclastogenesis.. J Biol Chem.

[22] Supek F, Supekova L, Mandiyan S, Pan YC, Nelson H (1994). A novel accessory subunit for vacuolar H(+)-ATPase from chromaffin granules.. J Biol Chem.

[23] Getlawi F, Laslop A, Schagger H, Ludwig J, Haywood J (1996). Chromaffin granule membrane glycoprotein IV is identical with Ac45, a membrane-integral subunit of the granule's H(+)-ATPase.. Neurosci Lett.

[24] Holthuis JC, Jansen EJ, Schoonderwoert VT, Burbach JP, Martens GJ (1999). Biosynthesis of the vacuolar H+-ATPase accessory subunit Ac45 in Xenopus pituitary.. Eur J Biochem.

[25] Muench SP, Huss M, Song CF, Phillips C, Wieczorek H (2009). Cryo-electron microscopy of the vacuolar ATPase motor reveals its mechanical and regulatory complexity.. J Mol Biol.

[26] Wilkens S, Forgac M (2001). Three-dimensional structure of the vacuolar ATPase proton channel by electron microscopy.. J Biol Chem.

[27] Jansen EJ, Holthuis JC, McGrouther C, Burbach JP, Martens GJ (1998). Intracellular trafficking of the vacuolar H+-ATPase accessory subunit Ac45.. J Cell Sci.

[28] Louagie E, Taylor NA, Flamez D, Roebroek AJ, Bright NA (2008). Role of furin in granular acidification in the endocrine pancreas: identification of the V-ATPase subunit Ac45 as a candidate substrate.. Proc Natl Acad Sci U S A.

[29] Blair HC, Teitelbaum SL, Ghiselli R, Gluck S (1989). Osteoclastic bone resorption by a polarized vacuolar proton pump.. Science.

[30] Camacho M, Machado JD, Montesinos MS, Criado M, Borges R (2006). Intragranular pH rapidly modulates exocytosis in adrenal chromaffin cells.. J Neurochem.

[31] Gluck S, Cannon C, Al-Awqati Q (1982). Exocytosis regulates urinary acidification in turtle bladder by rapid insertion of H+ pumps into the luminal membrane.. Proc Natl Acad Sci U S A.

[32] Palmgren MG (1991). Acridine orange as a probe for measuring pH gradients across membranes: mechanism and limitations.. Anal Biochem.

[33] Baron R (1989). Molecular mechanisms of bone resorption by the osteoclast.. Anat Rec.

[34] Vaananen HK, Zhao H, Mulari M, Halleen JM (2000). The cell biology of osteoclast function.. J Cell Sci.

[35] Bayer MJ, Reese C, Buhler S, Peters C, Mayer A (2003). Vacuole membrane fusion: V0 functions after trans-SNARE pairing and is coupled to the Ca2+-releasing channel.. J Cell Biol.

[36] Ochotny N, Flenniken AM, Owen C, Voronov I, Zirngibl RA (2011). The V-ATPase a3 subunit mutation R740S is dominant negative and results in osteopetrosis in mice.. J Bone Miner Res.

[37] Jansen EJ, Scheenen WJ, Hafmans TG, Martens GJ (2008). Accessory subunit Ac45 controls the V-ATPase in the regulated secretory pathway.. Biochim Biophys Acta.

[38] El Far O, Seagar M (2011). A role for V-ATPase subunits in synaptic vesicle fusion?. J Neurochem.

[39] Peters C, Bayer MJ, Buhler S, Andersen JS, Mann M (2001). Trans-complex formation by proteolipid channels in the terminal phase of membrane fusion.. Nature.

[40] Ishizuka H, Garcia-Palacios V, Lu G, Subler MA, Zhang H (2011). ADAM8 enhances osteoclast precursor fusion and osteoclast formation in vitro and in vivo.. J Bone Miner Res.

[41] Schoonderwoert VT, Martens GJ (2002). Targeted disruption of the mouse gene encoding the V-ATPase accessory subunit Ac45.. Mol Membr Biol.

[42] Crawford LW, Foley JF, Elmore SA (2010). Histology atlas of the developing mouse hepatobiliary system with emphasis on embryonic days 9.5-18.5.. Toxicol Pathol.

[43] Ramirez MI, Pollack L, Millien G, Cao YX, Hinds A (2002). The alpha-isoform of caveolin-1 is a marker of vasculogenesis in early lung development.. J Histochem Cytochem.

[44] Jansen EJ, Hafmans TG, Martens GJ (2010). V-ATPase-mediated granular acidification is regulated by the V-ATPase accessory subunit Ac45 in POMC-producing cells.. Mol Biol Cell.

[45] Drose S, Altendorf K (1997). Bafilomycins and concanamycins as inhibitors of V-ATPases and P-ATPases.. J Exp Biol.

[46] Xu J, Feng HT, Wang C, Yip KH, Pavlos N (2003). Effects of Bafilomycin A1: an inhibitor of vacuolar H (+)-ATPases on endocytosis and apoptosis in RAW cells and RAW cell-derived osteoclasts.. J Cell Biochem.

[47] Roodman GD (1999). Cell biology of the osteoclast.. Exp Hematol.

[48] Nesbitt SA, Horton MA (1997). Trafficking of matrix collagens through bone-resorbing osteoclasts.. Science.

[49] Salo J, Lehenkari P, Mulari M, Metsikko K, Vaananen HK (1997). Removal of osteoclast bone resorption products by transcytosis.. Science.

[50] Feng S, Deng L, Chen W, Shao J, Xu G (2009). Atp6v1c1 is an essential component of the osteoclast proton pump and in F-actin ring formation in osteoclasts.. Biochem J.

[51] Vitavska O, Merzendorfer H, Wieczorek H (2005). The V-ATPase subunit C binds to polymeric F-actin as well as to monomeric G-actin and induces cross-linking of actin filaments.. J Biol Chem.

[52] Vitavska O, Wieczorek H, Merzendorfer H (2003). A novel role for subunit C in mediating binding of the H+-V-ATPase to the actin cytoskeleton.. J Biol Chem.

[53] Yagi M, Miyamoto T, Sawatani Y, Iwamoto K, Hosogane N (2005). DC-STAMP is essential for cell-cell fusion in osteoclasts and foreign body giant cells.. J Exp Med.

[54] Lewandoski M (2001). Conditional control of gene expression in the mouse.. Nat Rev Genet.

[55] Meyers EN, Lewandoski M, Martin GR (1998). An Fgf8 mutant allelic series generated by Cre- and Flp-mediated recombination.. Nat Genet.

[56] Gu H, Marth JD, Orban PC, Mossmann H, Rajewsky K (1994). Deletion of a DNA polymerase beta gene segment in T cells using cell type-specific gene targeting.. Science.

[57] Xu J, Tan JW, Huang L, Gao XH, Laird R (2000). Cloning, sequencing, and functional characterization of the rat homologue of receptor activator of NF-kappaB ligand.. J Bone Miner Res.

[58] Livak KJ, Schmittgen TD (2001). Analysis of relative gene expression data using real-time quantitative PCR and the 2(-Delta Delta C(T)) Method.. Methods.

[59] Pavlos NJ, Cheng TS, Qin A, Ng PY, Feng HT (2011). Tctex-1, a Novel Interaction Partner of Rab3d, Is Required for Osteoclastic Bone Resorption.. Mol Cell Biol.

