# Supplementary figures and images for: Versatile Roles of V-ATPases Accessory Subunit Ac45 in Osteoclast Formation and Function
Source: PLoS One. 2011 Nov 4;6(11):e27155. doi: 10.1371/journal.pone.0027155 (PMC3210823; doi:10.1371/journal.pone.0027155)

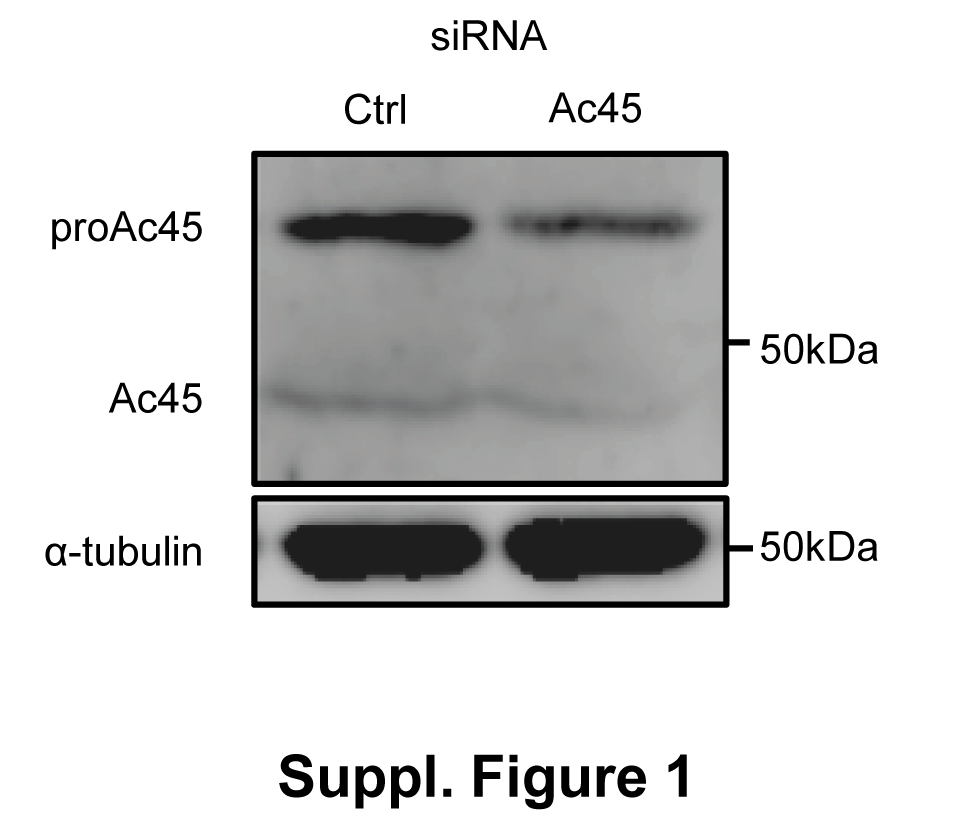

Supplement: Figure S1 — ProAc45 protein level following Ac45 silencing. Following 48hrs transfection with 100nM siRNA, total cellular proteins were extracted from transfected mBMM as described in Materials and Methods. Rabbit polyclonal anti-Ac45 antibody was used to detect proAc45 and mature Ac45 [24]. Ac45 is produced as a ∼62kDa precursor protein (proAc45) that is subsequently processed into the mature ∼45kDa form. The Ac45 siRNA appears to affect both the proAc45 and mature Ac45 protein levels. (TIF) [file pone.0027155.s001.tif]

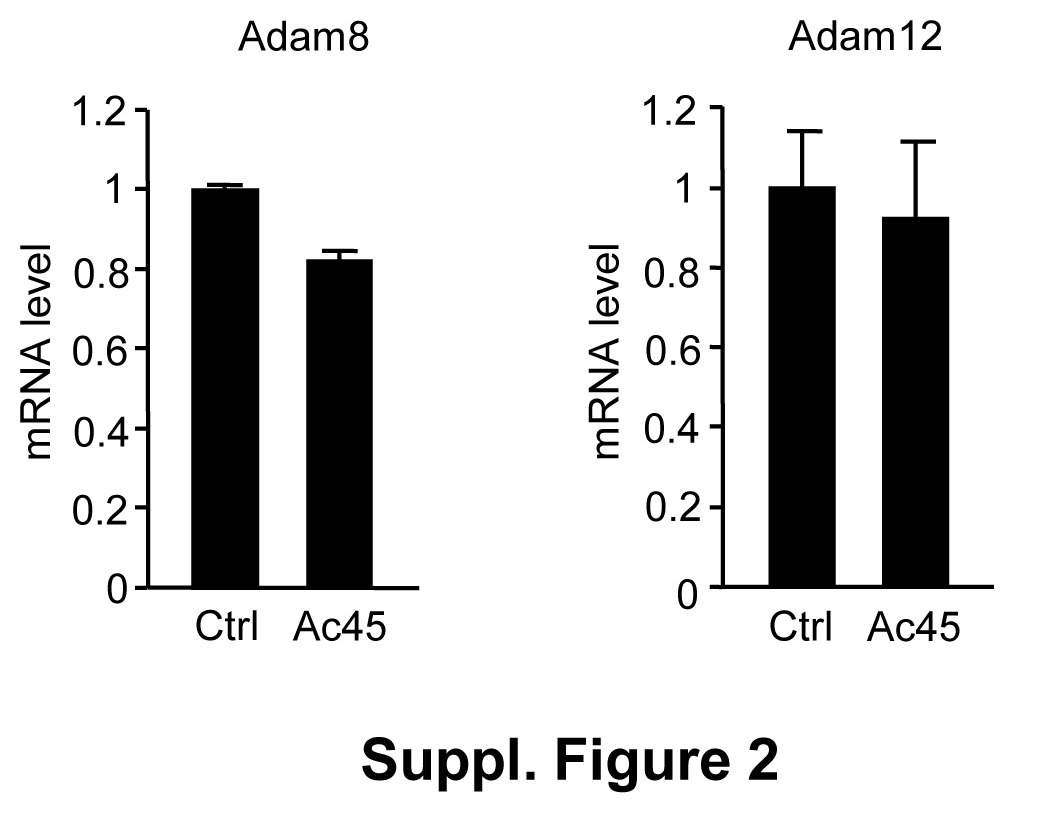

Supplement: Figure S2 — Quantitative analysis of Adam8 and Adam12 gene expression following Ac45 silencing using real-time qPCR. mRNA from pre-osteoclasts transfected with Ac45 siRNA for 48hrs was subjected to qPCR analysis using specific primers for ADAM8 and ADAM12. Data represented as relative mRNA level normalized to 36B4 control ± SEM (n = 3). (TIF) [file pone.0027155.s002.tif]

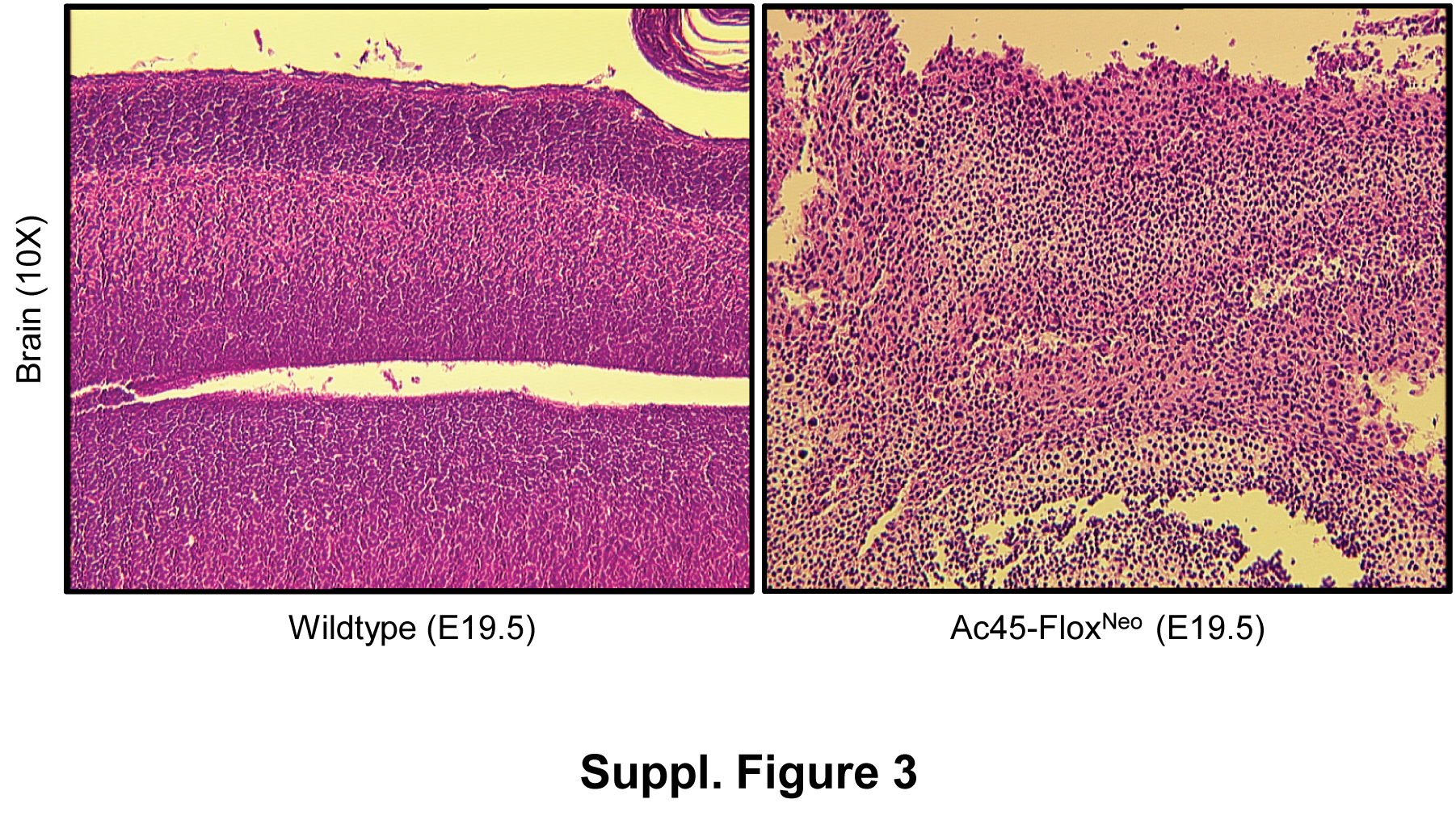

Supplement: Figure S3 — Comparison of WT wildtype E19.5 embryos and Ac45-FlexNeo embryo. H&E staining demonstrated that well-developed brain structure in the wildtype E19.5 embryos whilst the brain in Ac45-FlexNeo embryo development is severely impaired. (TIF) [file pone.0027155.s003.tif]
