# Supplementary material for: Versatile Roles of V-ATPases Accessory Subunit Ac45 in Osteoclast Formation and Function
Source: PLoS One. 2011 Nov 4;6(11):e27155. doi: 10.1371/journal.pone.0027155 (PMC3210823; doi:10.1371/journal.pone.0027155)
Supplement: Table S1 — Primers for Semi-quantitative and Real-time PCR. (DOCX) [file pone.0027155.s004.docx]

**Table S1:** **Primers for Semi-quantitative and Real-time PCR**

| Gene | Forward | Reverse |
| --- | --- | --- |
| For Semi-quantitative RT-PCR | | |
| Ac45  (ATP6AP1) | 5′-AGATCTACCATGATGGCGGCAACAGT-3′ | 5′-AGATCTTCCACAATCTGGGTCAAAGTGA-3′ |
| ATP6V0a3 | 5′-GGATCCGAATTCATCATGGGCTCTATGTTC-3′ | 5′-GGATCCTCTAGACTAGTCACTGTCCACAGT-3′ |
| ATP6V0d2 | 5′-GGATCCGAATTCATGCTTGAGACTGCAGAG-3′ | 5′-GGTCTAGATTATAAAATTGGAATGTAGCT-3′ |
| CTR | 5′-TGGTTGAGGTTGTGCCCA-3′ | 5′-CTCGTGGGTTTGCCTCATC-3′ |
| Cathepsin K | 5′-GGGAGAAAAACCTGAAGC-3′ | 5′-ATTCTGGGGACTCAGAGC-3′ |
| TRAP | 5′-CAGCAGCCAAGGAGGACTAC-3′ | 5′-ACATAGCCCACACCGTTCTC-3′ |
| 18S | 5′-ACCATAAACGATGCCGACT-3′ | 5′-TGTCAATCCTGTCCGTGTC-3′ |
| For Real-time qPCR | | |
| Ac45 | 5′-CCTGGAGCTGGGTCCCCGTAAT-3′ | 5′-GGGCCAAGTCCAGGGCATTCT-3′ |
| ATP6V0d2 | 5′-TTCCTTGGAGCCCCTGAGCACAT-3′ | 5′-TGTGAAACGGCCCAGTGGGTG-3′ |
| ATP6V0a3 | 5′-GCCTCAGGGGAAGGCCAGATCG-3′ | 5′-GGCCACCTCTTCACTCCGGAA-3′ |
| ATP6V0c | 5′-TTTTCGGTGTCATGGGCGCC-3′ | 5′-TGCGATGCCAGTGCCACTCTT-3′ |
| ATP6V0c″ | 5′-TTCTGGGCCTGCATGGTCGTT-3′ | 5′-ATGCCATAGATGGCCACCGCT-3′ |
| ATP6V1E1 | 5′-ACCCTGGAAAGCCGGCTGGA-3′ | 5′-ACAGCCGTGGGGGAACTGCT-3′ |
| ATP6V1B2 | 5′-ACGCTGAAGCGCTGCGAGAG-3′ | 5′-CCTTCCACTCGACCGGCACG-3′ |
| ADAM8 | 5′-CCACCAAGCCCCTCCCAGAGC-3′ | 5′-CCTCCAGTCCCGGGCTTGAC-3′ |
| ADAM12 | 5′-CACCATCGGCATGGCACCCA-3′ | 5′-TGCCAAGGTCACTGCGGCAC-3′ |
